# Younger adults are not alright, but older adults are? Examining mortality disparities among the children of migrants aged 15–44 and 45–64 in Sweden, 1990–2023: a total-population cohort study

**DOI:** 10.1136/bmjph-2025-003540

**Published:** 2026-03-26

**Authors:** Matthew Wallace

**Affiliations:** 1 Centre for Research on Inclusive Society, University of Salford, Salford, UK; 2 Stockholm University Demography Unit, Stockholm University, Stockholm, Sweden

**Keywords:** Sociodemographic Factors, Public Health, Epidemiology, Demography, Community Health

## Abstract

**Introduction:**

Younger working age children of migrants have higher mortality risks relative to younger working age non-migrants in Europe. Yet, these are unique ages of mortality where the absolute risk of death is low and driven by external causes (ie, accidents, injuries and suicides). It remains unclear whether this higher mortality risk presents among older working age children of migrants—ages at which the absolute risk of death begins to increase exponentially and becomes dictated by chronic morbidities. We aim to fill this gap.

**Methods:**

We fit survival models on Swedish total population register data using an extended, competing risks approach. We investigate all-cause and mortality from natural causes and external causes, drugs and alcohol among the children of migrants, migrants and non-migrants aged 15–44 and 45–64 from 1990 to 2023. We report both HRs and regression-standardised cumulative probabilities of death.

**Results:**

Younger working age children of migrants from an array of origins exhibit higher external, drug and alcohol mortality relative to non-migrants that drives their higher all-cause mortality risk. Older working age children of migrants from multiple origins also present with a higher risk of external, drug and alcohol mortality, but only exhibit comparable all-cause mortality to non-migrants, owing to their similar to lower mortality from natural causes. Disparities in socioeconomic background largely attenuate these risks. A diverse range of younger and older working age migrants exhibit lower all-cause, natural, and external, drug and alcohol mortality than non-migrants do.

**Conclusions:**

Interventions should be made to address the elevated avoidable and preventable external, drugs and alcohol mortality that presents among both older and younger working age children of migrants. Doing so will generate reductions in their all-cause mortality risk.

**WHAT IS ALREADY KNOWN ON THIS TOPIC**

- A review reports higher all-cause and external mortality among younger working age children of migrants in Europe—especially in relative terms. Socioeconomic disadvantage plays a key role in attenuating these disparities between the children of migrants and non-migrants. However, younger working ages are ages at which the absolute risk of death is low in high-income countries and driven by external causes of death like drug misuse and suicide. Findings for these ages cannot be generalised to older working age children of migrants, because these are ages at which mortality begins increasing exponentially and becomes dictated by chronic morbidities and natural causes-of-death.

**WHAT THIS STUDY ADDS**

- We know little about the mortality risks of older working age children of migrants and whether this population, like their young adult counterparts, exhibits important disparities in their all-cause and external mortality risks relative to older working age non-migrants. We report a difference in all-cause mortality risks: younger working age adult children of migrants report higher mortality risks than younger working age non-migrants, while older working age children of migrants report comparable mortality to older working age non-migrants. Despite this, elevated external causes, drugs and alcohol mortality remains a prominent feature of both of these subpopulations.

**HOW THIS STUDY MIGHT AFFECT RESEARCH, PRACTICE OR POLICY**

- We call for interventions among the children of migrants to address their higher risks of preventable and avoidable external, drug and alcohol death at younger and older working ages. Policy makers could look to address socioeconomic factors that are strongly linked with external causes of death, including poverty, inequality and a lack of opportunity, neighbourhood segregation and isolation. Community and family-centred programmes that seek to address mental health, substance and alcohol use, violence intervention and mediation, and peer mentoring could also be implemented.

## Introduction

 A recent review of mortality among the children of migrants in Europe reports higher adult mortality risks relative to migrants with the same origins and non-migrants^1^—notably at younger working ages and among the children of migrants with parents born in low and middle-income countries.^1^ External causes of death, including suicide, substance misuse and accidents and injuries, have been identified as potentially salient causes of death—although evidence remains scarce.^1^ What’s more, large socioeconomic inequalities often attenuate the increased mortality risks of the children of migrants.^1^ This contrasts with migrants, for whom reviews report globally lower mortality among migrants relative to non-migrants, including for all major International Classification of Diseases (ICD) groups—except communicable diseases.^2^,^5^ Mortality is lowest among young adult migrants and those born in low-income and middle-income countries that move to high-income countries.^2 4 5^ The lower mortality risks of international migrants often belie their lower socioeconomic status.^6^

So far, the studies of mortality among the children of migrants (which typically include migrants) have focused on differences in mortality to non-migrants at younger working ages,^17^,^10^ or across the entire working age range.^11^,^14^ Consequently, these studies either narrow in on a unique age range of mortality where the absolute risk of death remains low and dictated by external causes like accidents, injuries and suicides (the mortality ‘accident hump’),^15^ or completely encompass the transition from ‘accident hump’ to the onset of an exponentially increasing risk of death driven by chronicity, morbidity and biological ageing. It remains unclear exactly what the mortality situation of older working age children of migrants—a population that has grown steadily in Europe in recent years^16 17^—looks like, and whether or not this group encounters the same risks as their younger peers.

We aim to understand whether the higher all-cause and external mortality risks reported in younger working age children of migrants are present among older working age children of migrants too. We fit extended multistate flexible survival models on a large cohort of total population administrative register data from Sweden from 1990 to 2023. We report both HRs (a relative measure) and regression-standardised cumulative probabilities of death (an absolute measure) across a diverse range of adult children of migrant populations aged 15–44 and 45–64, and include their counterpart migrant groups for additional insight. We make calls for specific interventions based on our findings.

## Data and methods

We used the collection of Swedish register data REFU-GEN at Stockholm University. This collection covers longitudinal, individual-level data from a range of administrative sources. The analysis was performed under an existing ethical approval provided by the Swedish Ethical Review Authority. REFU-GEN has been generated and pseudonymised by Statistikmyndigheten (SCB Statistics Sweden) for the purposes of academic research.

To be eligible for the study, people had to be (a) registered as resident in Sweden for at least 1 year between 1990 and 2023 and (b) contribute to the age range 15 and 64 years old during that period. A single dataset was built and the episode was split into age groups 15–44 years and 45–64 years. Consistent with prior research on mortality among children of migrants, we focus on mortality at working ages as a clear age segment conceptually associated with premature and avoidable mortality,^13^ because these are the ages at which the children of migrants are most intensely concentrated in Sweden,^16^ because these are the ages which prior research on this population has been carried out,^17^,^13^ and because these are the ages at which death is most sensitive to inequalities in social conditions.^18^

The outcome in the models was all-cause mortality and death from natural (all ICD-9 codes 0–799; all ICD-10 codes A00–R98) and external, drug and alcohol-related death (ICD9-codes 291, 292, 303–305, 357.5, 425.5, 535.3, 571.0–571.3, 655.4, 760.71, 800–999; ICD-10 codes F10–F16, F18–F19, G62.1, G31.2, G72.1, I42.6, K29.2, K70.0-K70.4, K70.9, K85.2, K86.0, Q86.0, PO4.3, V00–Y99). Mortality from ill-defined causes (ICD9-codes 797–799 and ICD-10 codes R00–R99), which only account for a small percentage of all deaths at ages 15–44 years (2138; 3.0%) and 45–64 years (6669; 2.1%), is included in both the analyses of all-cause and cause-specific mortality (as a competing risk) but is not presented in the primary results. Instead, a supplementary analysis of ill-defined causes of death is available as part of the online materials.

Exposure was generational status by country/region of birth. We classified individuals as part of the majority population if they were born in Sweden to two parents who were also born in Sweden. We classified individuals as ‘migrants’ if they were born outside of Sweden. We classified individuals as ‘children of migrants’ if they were born in Sweden to at least one parent born outside of Sweden. We disaggregated generations by country/region of individual and parental origins into Finland, other Nordic countries, former Yugoslavia, Baltic States, DACH (Germany, Austria and Switzerland), USA, UK, Canada, Australia (Aus) and New Zealand (NZ), other Western Europe, other Central & Eastern Europe, Central & Southern America, Middle East, North Africa and Turkey (MENAT), Sub-Saharan Africa and Asia. These origin groups broadly reflect the migration history of Sweden in the second half of the 20th century.^19^

Predictor variables included sex, birth year, civil status, highest level of education and disposable income. Sex was categorised into ‘male’ and ‘female’. Civil status was grouped into ‘single’, ‘married’, ‘divorced’ and ‘widowed’ and included same sex partnerships. Education was coded using the International Standard Classification of Education (ISCED) into ‘primary’, ‘secondary’ and ‘post-secondary’ education. Disposable income was based on a variable that records individuals’ disposable income for each calendar year. We generated annual quintiles of disposable income that ranged from ‘lowest’ (Q1), ‘lower’, ‘medium’, ‘higher’ to ‘highest’ (Q5). We permitted people’s values for civil status, education and disposable income to time-vary every 3 years.

We conducted a complete case analysis—an analysis that includes only individuals for which there is no missing information in the exposure or predictor variables. A complete case analysis may be used as the primary analysis if the proportions of missing data are below 5%.^20^ Here, we dropped 0.8% of episodes at ages 15–44 and 0.1% of episodes at ages 45–64 due to missing information in individual and/or parental country of birth, education, disposable income and civil status—retaining over 99% for a final analysis.

All of the models were fitted with flexible parametric survival models ‘stpm3’ in Stata V.18,^21 22^ episode splitting by age (into 15–44 and 45–64), and using the extended, multistate competing risks approach outlined in Putter *et al*.^23^ We reported the HRs alongside their 95% confidence limits and generated regression-standardised cumulative probabilities of death using ‘standsurv’ from Stata V.18. In each model, the HRs represent the ratio of the hazard rate for all-cause or cause-specific mortality among migrants and the children of migrants as compared with the respective hazard rate of the majority population, before and after adjusting for specified controls.

All estimates are derived from two models: model 1 (ages 15–44) and model 2 (ages 45–64). For model 1, individuals joined the risk set in 1990 so long as they were resident in Sweden (using an annual indicator of residence) at the end of 1989 and aged between 15 and 44 on 1 January 1990. Individuals could also join the risk set during the analysis period on turning 15 years old (having been born in Sweden or arrived in Sweden as a migrant younger than age 15 in 1990) and by arriving in Sweden from another country if aged between 15 and 44. Individuals exited the risk set if they died before age 44, emigrated before age 44, reached age 44 before the end of the analysis period on 31 December 2023, or were alive and resident in Sweden on 31 December 2023 and aged between 15 and 44. The same logic with age parameters 45 and 64 applies to model 2.

We fitted partially adjusted models and fully adjusted models for all-cause and cause-specific mortality. The partially adjusted model adjusted for birth year (continuous), sex (with female as the reference), and target population (with the non-migrant population as the reference). The fully adjusted model additionally controlled for the highest level of education (with post-secondary education as the reference), disposable income (with the highest income quintile as the reference), and civil status (married as the reference).

We conducted additional analyses. Online supplemental file 1 provides an analysis of ill-defined causes of death, online supplemental file 2 shows the analysis from the main paper stratified by sex, and online supplemental file 3 provides the distributions of education level and disposable income for the specific migrant and children of migrant subgroups.

## Results

Table 1 reports the person-years at risk and number of all-cause, natural and external, drug and alcohol deaths among migrants and the children of migrants at ages 15–44 and 45–64 years old by generation and specific migrant origins. We provide this numerator and denominator information to aid interpretation of the HRs and regression-standardised probabilities of death. In total, we examine 69 837 deaths and 126 036 796 person-years at ages 15–44 and 310 429 deaths and 78 929 933 person-years at ages 45–64.

**Table 1:** Person-years and the number of deaths by cause at younger and older adult working ages among migrants and children of migrants living in Sweden, 1990–2023

Population	15–44 years old				45–64 years old			
	Person-years at risk	All-cause deaths	Natural deaths	External, drugs and alcohol deaths	Person-years at risk	All-cause deaths	Natural deaths	External, drugs and alcohol deaths
Non-migrants	86 453 116	48 611	23 927	23 609	61 002 316	245 348	214 666	26 915
Migrants	24 282 869	11 225	5646	4889	12 867 321	47 418	40 114	4845
Finland	2 583 795	2025	1046	935	2 827 599	16 572	14 060	2033
Other Nordic countries	1 176 449	584	284	272	961 536	4504	3915	448
Former Yugoslavia	2 529 568	1072	574	426	1 477 271	5389	4603	359
DACH countries	599 464	208	103	95	564 363	2258	1920	230
Baltic States	255 741	81	35	38	130 722	585	517	52
Other Western Europe	999 985	325	193	100	547 306	1702	1443	146
Other Eastern Europe	2 340 094	1157	551	487	1 377 815	4673	3768	574
UK, USA, Can, Aus, NZ	759 340	293	156	117	378 215	1069	912	126
Central South America	1 483 867	652	305	320	652 064	1395	1163	126
MENAT countries	6 786 610	2592	1218	1204	2 612 134	6026	5070	502
Sub-Saharan Africa	2 007 035	1182	637	445	526 983	1540	1272	130
Other non-Western	2 760 921	1054	544	450	811 313	1705	1471	119
Children of migrants	15 300 812	10 001	3900	5728	5 060 296	17 663	14 589	2631
Finland	4 965 334	4233	1638	2481	1 825 916	6435	5178	1079
Other Nordic countries	1 994 368	1420	638	753	1 306 003	5298	4492	699
Former Yugoslavia	1 104 111	544	204	310	108 138	209	159	44
DACH countries	1 161 445	746	350	371	658 436	1957	1633	279
Baltic States	251 112	219	128	83	250 774	846	719	105
Other Western Europe	874 780	440	175	244	197 978	491	401	74
Other Eastern Europe	1 088 055	639	225	383	341 597	1263	1048	180
UK, USA, Can, Aus, NZ	588 980	338	141	171	265 241	922	771	131
Central South America	561 672	237	65	156	21 137	57	45	11
MENAT countries	1 716 343	733	210	482	38 220	77	57	12
Sub-Saharan Africa	380 508	199	48	136	14 129	31	18	9
Other non-Western	614 103	253	78	158	32 729	77	68	8
Total	126 036 796	69 837	33 473	34 226	78 929 933	310 429	269 369	34 391

Source: author’s calculations based on Swedish register data collection REFU-GEN. Notes: The small difference in the sum of natural and external, drug and alcohol-related causes relative to all-cause deaths reflects the number of deaths from ill-defined causes.

Aus, Australia; Can, Canada; DACH, Germany, Austria and Switzerland; MENAT, Middle East, North Africa and Turkey; NZ, New Zealand.

**Figure 1:**
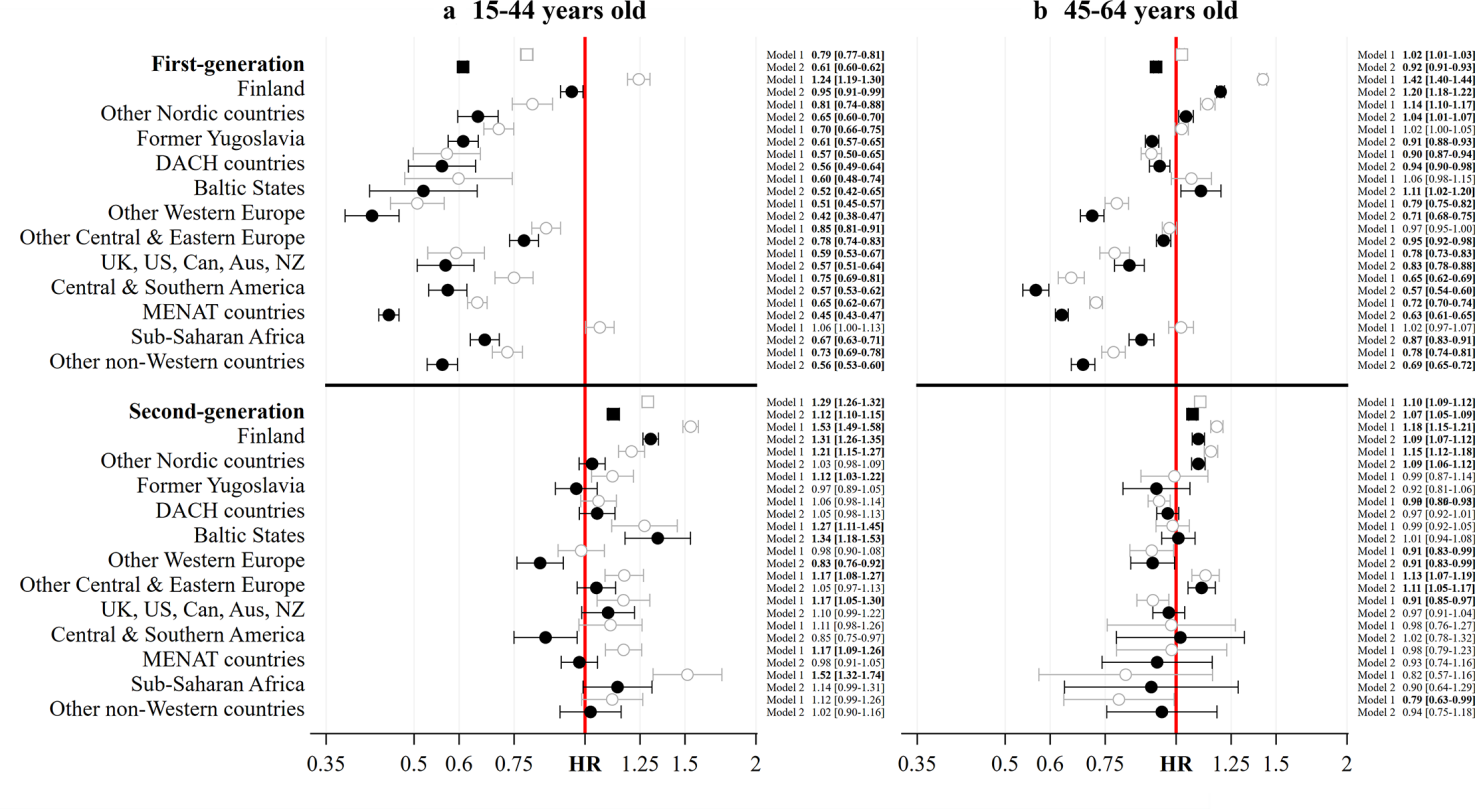
HRs of all-cause mortality among migrants and children of migrants aged 15–44 and 45–64 in Sweden, 1990–2023, before and after adjusting for differences in demographic and socioeconomic background. Source: author’s calculations based on Swedish register data collection REFU-GEN. Notes: Minimally adjusted ‘Min. Adj.’ model=age+birth year+sex+target population. Fully adjusted ‘Full. Adj.’ model =the minimally adjusted model+civil status+education+disposable income. Aus, Australia; Can, Canada; DACH, Germany, Austria and Switzerland; MENAT, Middle East, North Africa and Turkey; NZ, New Zealand.

Figure 1 displays HRs and adjusted HRs (aHRs) for all-cause mortality. We report evidence of higher mortality risks among younger working age children of migrants in panel (a) of of figure 1. Before adjustment for differences in socioeconomic background, all groups have higher mortality compared with non-migrants, except DACH countries, other Western Europe, Central & Southern America, and other non-Western countries. HRs are highest among children of migrants with parent(s) born in Finland (HR=1.53 (95% CIs 1.49 to 1.58)) and Sub-Saharan Africa (HR=1.52 (95% CIs 1.32 to 1.74)). After adjustment for socioeconomic status, the higher mortality is attenuated in all groups except Finland (aHR=1.31 (95% CIs 1.26 to 1.35)) and Baltic States (aHR=1.34 (95% CIs 1.18 to 1.53)). Among older working age children of migrants in panel (b) of figure 1, we only report persistent evidence of higher mortality among those with parent(s) born in Finland (aHR=1.09 (95% CIs 1.07 to 1.12)), the other Nordic countries (aHR=1.09 (95% CIs 1.07 to 1.12)) and other Central & Eastern Europe (aHR=1.11 (95% CIs 1.05 to 1.17)). No other groups display evidence of a higher mortality risk compared with non-migrants—either before or after adjustment for socioeconomic status.

Staying with figure 1 and panel (a), we find widespread evidence of lower all-cause mortality among younger working age migrants before adjustment for socioeconomic background except among migrants born in Finland (HR=1.24 (95% CIs 1.19 to 1.30)) and in Sub-Saharan Africa (HR=1.06 (95% CIs 1.00 to 1.13)). After this adjustment, all younger working age migrant groups display lower mortality risks than non-migrants. Among older working age migrants in panel (b) of figure 1, there continues to be evidence of lower mortality in six migrant populations before and after adjustment for socioeconomic background, including other Western Europe (aHR=0.71 (95% CIs 0.68 to 0.75)); the UK, USA, Can, Aus and NZ (aHR=0.83 (95% CIs 0.78 to 0.88)), Central & Southern America (aHR=0.83 (95% CIs 0.78 to 0.88)); MENAT (aHR=0.57 (95% CIs 0.54 to 0.60)); Sub-Saharan Africa (aHR=0.87 (95% CIs 0.83 to 0.91)), and other non-Western countries (aHR=0.69 (95% CIs 0.65 to 0.72)). The other six populations report HRs and aHRs closer to or above a HR of one.

**Figure 2:**
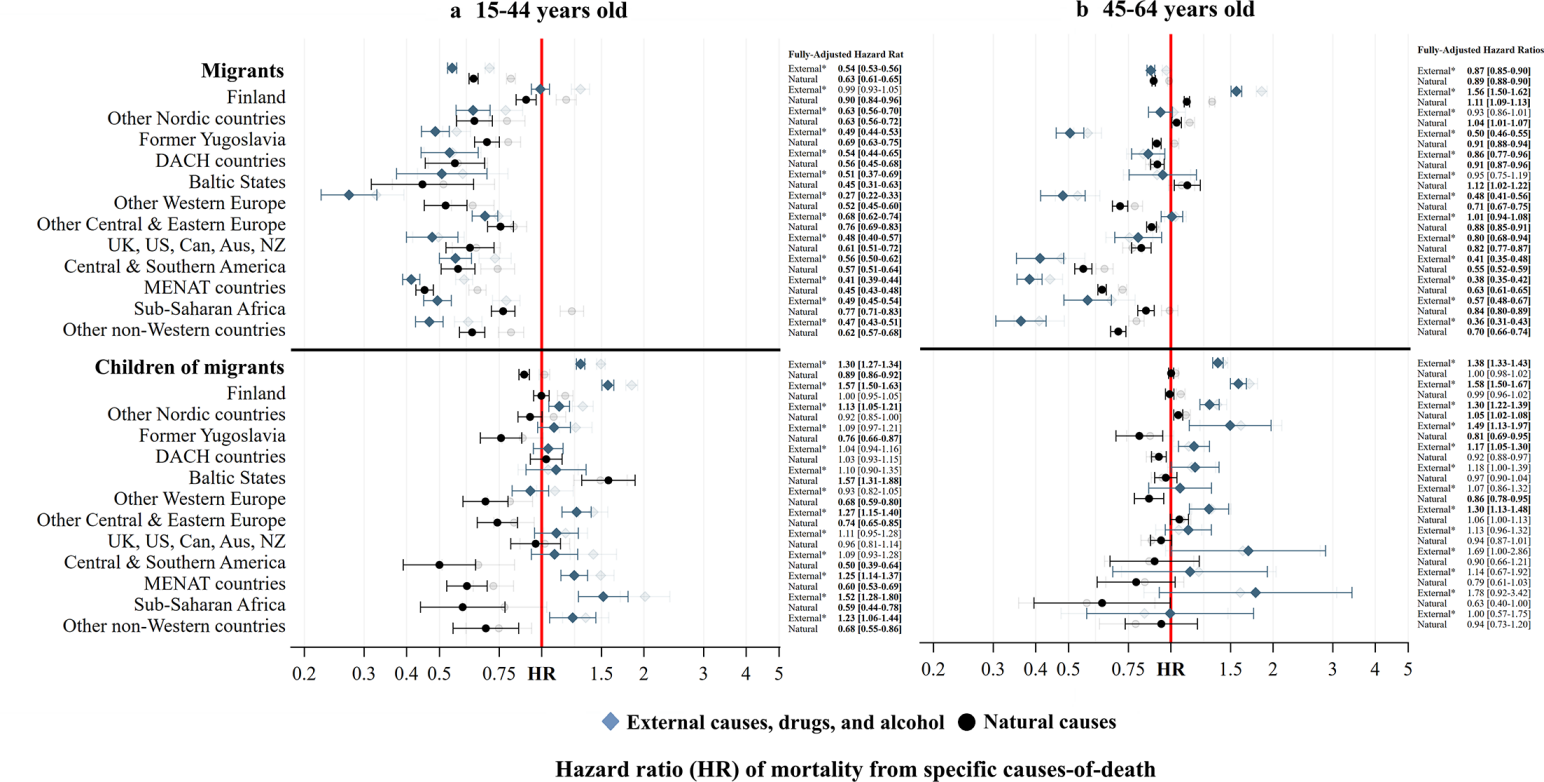
HRs of external, drug, alcohol and natural mortality among migrants and children of migrants aged 15–44 and 45–64 in Sweden, 1990–2023, after adjusting for demographic and socioeconomic background. Source: author’s calculations based on Swedish register data collection REFU-GEN. Notes: Fully adjusted ‘Full. Adj.’ model=age+birth year+sex+target population+civil status+education level+disposable income. External* refers to mortality from external causes, drug and alcohol. The greyed out estimates refer to the HRs from the minimally adjusted models. Aus, Australia; Can, Canada; DACH, Germany, Austria and Switzerland; MENAT, Middle East, North Africa and Turkey; NZ, New Zealand.

Figure 2 displays the fully adjusted aHRs for cause-specific mortality, with minimally adjusted HRs provided ‘greyed’ out in the background for reference. Among younger working age (panel (a)) and older working age (panel (b)) children of migrants, mortality from external causes, drugs and alcohol is high in all groups except for younger and older working age migrants born in other Western Europe, DACH countries, UK, USA, Can, Aus and NZ and older working age children of migrants born in other non-Western countries. Even in the fully adjusted model, aHRs remain highly elevated among younger working age children of migrants with parent(s) born in Finland (aHR=1.57 (95% CIs 1.50 to 1.63)), other Nordic countries (aHR=1.13 (95% CIs 1.05 to 1.21)), other Central & Eastern Europe (aHR=1.57 (95% CIs 1.31 to 1.88)), MENAT (aHR=1.25 (95% CIs 1.14 to 1.37)), Sub-Saharan Africa (aHR=1.52 (95% CIs 1.28 to 1.80)) and other non-Western countries (aHR=1.23 (95% CIs 1.06 to 1.44)), and among older working age children of migrants with parent(s) born in Finland (aHR=1.58 (95% CIs 1.50 to 1.67)), other Nordic countries (aHR=1.30 (95% CIs 1.22 to 1.39)), former Yugoslavia (aHR=1.49 (95% CIs 1.13 to 1.97)), DACH (aHR=1.17 (95% CIs 1.05 to 1.30)) and other Central & Eastern Europe (aHR=1.30 (95% CIs 1.13 to 1.48)). In contrast, mortality from natural causes is typically similar to or lower than it is among non-migrants. Only younger working age children of migrants with parent(s) born in Baltic States (aHR=1.57 (95% CIs 1.31 to 1.88)) and older working age migrants with parent(s) born in other Nordic countries (aHR=1.05 (95% CIs 1.02 to 1.08)) report a higher risk of natural mortality compared with the non-migrant population.

Remaining with figure 2 and panel (a), all younger working age migrants have lower mortality from natural and external causes compared with non-migrants, except migrants born in Finland, who have comparable external, drug and alcohol mortality to the majority population. Older working age migrants (in panel (b)) born in other Western Europe, the UK, USA, Can, Aus and NZ, Central & Southern America, MENAT countries, Sub-Saharan Africa and other non-Western countries, like their younger working age counterparts, display lower external, drug, alcohol and natural mortality compared with non-migrants. Both natural (aHR=1.11 (95% CIs 1.09 to 1.13)) and external, drug and alcohol mortality (aHR=1.56 (95% CIs 1.50 to 1.62)) are raised among migrants born in Finland. Natural mortality is raised for migrants born in Baltic States (aHR=1.12 (95% CIs 1.02 to 1.22)).

Table 2 shows how the aHRs in figures1  2 translate to absolute differences through regression standardised cumulative probabilities (SP) of all-cause and cause-specific mortality by age 44 and 64. For the total sample, we report a cumulative probability of death by age 44 of SP=2.2 (95% CIs 2.1 to 2.2) and age 64 of SP=8.3 (95% CIs 8.2 to 8.3). Put another way, we might expect 22 people to have died per 1000 people by age 44 years and 83 people to have died per 1000 people by age 64 between 1990 and 2023.

**Table 2:** Regression-standardised cumulative probabilities of death by ages 44–64

Population	15–44 years old			45–64 years old		
	All causes	External, drugs and alcohol	Natural causes	All causes	External, drugs and alcohol	Natural causes
	SP (95% CIs)	SP (95% CIs)	SP (95% CIs)	SP (95% CIs)	SP (95% CIs)	SP (95% CIs)
Non-migrants	2.3 (2.2 to 2.3)	1.2 (1.0 to 1.4)	1.0 (1.0 to 1.1)	8.5 (8.4 to 8.5)	1.3 (1.1 to 1.4)	7.1 (7.0 to 7.2)
First-generation	1.4 (1.3 to 1.4)	0.6 (0.5 to 0.8)	0.7 (0.6 to 0.7)	7.8 (7.6 to 8.0)	1.1 (0.8 to 1.5)	6.3 (6.2 to 6.4)
Finland	2.2 (2.1 to 2.3)	1.2 (1.0 to 1.4)	0.9 (0.9 to 1.0)	10.1 (9.9 to 10.4)	2.0 (1.7 to 2.3)	7.9 (7.6 to 8.2)
Other Nordic countries	1.5 (1.3 to 1.6)	0.7 (0.6 to 0.9)	0.7 (0.6 to 0.8)	8.8 (8.5 to 9.1)	1.2 (0.9 to 1.4)	7.4 (7.0 to 7.8)
Former Yugoslavia	1.4 (1.3 to 1.5)	0.6 (0.5 to 0.7)	0.7 (0.7 to 0.8)	7.7 (7.3 to 8.1)	0.6 (0.5 to 0.8)	6.4 (6.0 to 6.9)
DACH countries	1.3 (1.1 to 1.5)	0.6 (0.5 to 0.8)	0.6 (0.5 to 0.7)	7.9 (7.6 to 8.3)	1.1 (0.8 to 1.4)	6.5 (6.0 to 7.0)
Baltic States	1.2 (0.9 to 1.5)	0.6 (0.4 to 0.8)	0.5 (0.3 to 0.7)	9.4 (8.8 to 9.9)	1.2 (0.7 to 1.7)	7.9 (7.1 to 8.8)
Other Western Europe	1.0 (0.8 to 1.1)	0.3 (0.2 to 0.4)	0.5 (0.5 to 0.6)	6.0 (5.5 to 6.5)	0.6 (0.4 to 0.8)	5.0 (4.4 to 5.7)
Other Eastern Europe	1.8 (1.7 to 1.9)	0.8 (0.7 to 1.0)	0.8 (0.7 to 0.9)	8.0 (7.7 to 8.4)	1.3 (1.0 to 1.5)	6.2 (5.8 to 6.7)
USA, UK, Can, Aus, NZ	1.3 (1.1 to 1.5)	0.6 (0.4 to 0.7)	0.6 (0.5 to 0.8)	7.0 (6.4 to 7.6)	1.0 (0.7 to 1.3)	5.8 (5.0 to 6.6)
Central South America	1.3 (1.2 to 1.4)	0.7 (0.6 to 0.8)	0.6 (0.5 to 0.7)	4.8 (4.2 to 5.5)	0.5 (0.3 to 0.7)	3.9 (3.2 to 4.6)
MENAT countries	1.0 (1.0 to 1.1)	0.5 (0.4 to 0.6)	0.5 (0.4 to 0.5)	5.3 (4.9 to 5.7)	0.5 (0.4 to 0.6)	4.4 (4.0 to 4.9)
Sub-Saharan Africa	1.5 (1.4 to 1.6)	0.6 (0.5 to 0.7)	0.8 (0.7 to 0.9)	7.4 (6.3 to 8.4)	0.7 (0.5 to 1.0)	6.0 (4.9 to 7.1)
Other non-Western	1.3 (1.2 to 1.4)	0.5 (0.5 to 0.7)	0.7 (0.6 to 0.7)	5.8 (5.1 to 6.6)	0.5 (0.3 to 0.7)	5.0 (4.2 to 5.8)
Second-generation	2.6 (2.5 to 2.6)	1.5 (1.3 to 1.8)	0.9 (0.9 to 1.0)	9.1 (8.6 to 9.5)	1.7 (1.3 to 2.2)	7.1 (7.0 to 7.2)
Finland	3.0 (2.9 to 3.1)	1.8 (1.6 to 2.1)	1.0 (1.0 to 1.1)	9.3 (8.7 to 9.8)	2.0 (1.7 to 2.3)	7.0 (6.5 to 7.6)
Other Nordic countries	2.3 (2.2 to 2.5)	1.3 (1.1 to 1.6)	1.0 (0.9 to 1.1)	9.3 (8.8 to 9.8)	1.6 (1.4 to 1.9)	7.5 (6.9 to 8.0)
Former Yugoslavia	2.2 (2.0 to 2.4)	1.3 (1.1 to 1.5)	0.8 (0.6 to 1.0)	7.8 (4.0 to 12.3)	1.9 (1.0 to 3.1)	5.7 (1.8 to 10.3)
DACH countries	2.4 (2.2 to 2.6)	1.2 (1.0 to 1.5)	1.1 (1.0 to 1.2)	8.2 (7.3 to 9.0)	1.5 (1.1 to 1.8)	6.5 (5.7 to 7.4)
Baltic States	3.0 (2.7 to 3.4)	1.3 (1.0 to 1.7)	1.6 (1.4 to 1.9)	8.5 (7.3 to 9.9)	1.5 (1.0 to 2.0)	6.8 (5.6 to 8.2)
Other Western Europe	1.9 (1.7 to 2.1)	1.1 (0.9 to 1.3)	0.7 (0.6 to 0.9)	7.7 (5.9 to 9.7)	1.3 (0.8 to 2.0)	6.1 (4.2 to 8.1)
Other Eastern Europe	2.4 (2.2 to 2.6)	1.5 (1.3 to 1.8)	0.8 (0.6 to 0.9)	9.4 (8.3 to 10.5)	1.6 (1.2 to 2.1)	7.5 (6.4 to 8.7)
USA, UK, Can, Aus, NZ	2.5 (2.2 to 2.8)	1.3 (1.1 to 1.6)	1.0 (0.8 to 1.2)	8.2 (7.3 to 9.1)	1.4 (1.0 to 1.9)	6.6 (5.7 to 7.7)
Central South America	1.9 (1.6 to 2.3)	1.3 (1.1 to 1.6)	0.5 (0.3 to 0.8)	8.6 (3.9 to 14.1)	2.1 (0.5 to 4.9)	6.3 (1.5 to 12.3)
MENAT countries	2.2 (2.0 to 2.4)	1.5 (1.3 to 1.7)	0.6 (0.5 to 0.8)	7.8 (2.4 to 14.6)	1.4 (0.5 to 3.5)	5.6 (2.4 to 12.7)
Sub-Saharan Africa	2.6 (2.1 to 3.1)	1.8 (1.5 to 2.2)	0.6 (0.3 to 1.1)	7.7 (2.0 to 15.0)	2.2 (0.2 to 6.3)	4.4 (1.5 to 12.2)
Other non-Western	2.3 (2.0 to 2.7)	1.4 (1.2 to 1.8)	0.7 (0.4 to 1.1)	8.0 (4.2 to 12.4)	1.2 (0.2 to 3.2)	6.6 (2.7 to 11.3)
Total population	2.2 (2.1 to 2.2)	1.1 (1.0 to 1.4)	0.9 (0.9 to 1.0)	8.3 (8.2 to 8.3)	1.3 (1.1 to 1.4)	7.0 (6.9 to 7.1)

Regression-standardised cumulative death probabilities are derived from fully adjusted model = age + sex + birth year + civil status + education level + disposable income; the difference in the sum of the regression-standardised probabilities of death from natural and external, drug and alcohol compared with from all-cause mortality gives the value of the regression-standardised probability of mortality from ill-defined causes.

Source: author’s calculations based on Swedish register data collection REFU-GEN.

Aus, Australia; Can, Canada; DACH, Germany, Austria and Switzerland; MENAT, Middle East, North Africa and Turkey; NZ, New Zealand; SP, cumulative probabilities.

There are two main points to take away from table 2. First, although we report elevated mortality risks among young working age children of migrants in figure 1, these only translate to small absolute differences in the cumulative probability of death by age 44 years. Among the children of migrants with parent(s) born in Finland who reported the highest aHR of all young working age children of migrants from figure 1 (aHR=1.31 (95% CIs 1.26 to 1.35)), the difference in absolute terms is just ΔSP=0.4 (ie, SP=3.0 (95% CIs 2.9 to 3.1) vs SP=2.6 (95% CIs 2.5 to 2.6) in the majority population). This equates to four additional deaths per 1000 people by age 44 relative to non-migrants. Equally, the same also applies to the lower HRs of mortality reported among migrants. For MENAT migrants, who reported an aHR of just aHR=0.45 (95% CIs 0.43 to 0.47), this equates to 13 fewer deaths per 1000 people by age 44 when compared with the majority population (SP=1.0 (95% CIs 1.0 to 1.1) vs SP=2.3 (95% CIs 2.2 to 2.3)). The absolute differences are somewhat larger at older working ages where, for example, migrants born in Finland (SP=10.1 (95% CIs 9.9 to 10.4)) can expect to experience 16 additional deaths per 1000 people by age 64 compared with older working age non-migrants (SP=8.5 (95% CIs 8.4 to 8.5)); migrants born in MENAT nations (SP=5.3 (95% CIs 4.9 to 5.7)), in turn, might expect to experience 32 fewer deaths per 1000 people by age 64.

Second, the cumulative probabilities of death from table 2 help to explain why younger and older working age children of migrants exhibit the same cause-of-death risk patterns relative to the majority population (figure 2), but divergent all-cause mortality patterns (figure 1). At younger working ages, external causes, drugs and alcohol exert a greater weight in the cumulative probability of all-cause mortality for non-migrants, children of migrants and migrants compared with older working ages. In the total sample, half of the cumulative probability of all-cause mortality at younger working ages (SP=2.2 (95% CIs 2.1 to 2.2)) is attributable to external causes, drug and alcohol mortality alone (SP=1.1 (95% CIs 1.0 to 1.4)). At older working ages, where the cumulative probability of all-cause mortality is SP=8.3 (95% CIs 8.2 to 8.3), the contribution of external causes, drug and alcohol is considerably lower (SP=1.3 (95% CIs 1.1 to 1.4)), with natural causes accounting for the majority of risk at SP=7.0 (95% CIs 6.9 to 7.1)). These patterns apply across all subpopulations and become even more pronounced among the children of migrants. This shift in cause-of-death contributions explains the divergence in all-cause mortality differentials between the younger and older working age children of migrants.

### Supplementary analyses

Online supplemental file 1 investigates mortality from ill-defined causes. It shows that the percentage of ill-defined causes varies across the exposure and predictors (online supplemental table S1.1). It also shows that all migrant and children of migrant groups exhibit higher HRs of death from ill-defined causes (online supplemental table S1.2)—although this only translates to small cumulative probabilities of mortality by ages 44 and 64 (online supplemental table S1.2). Online supplemental table S1.3 (15–44) and online supplemental table S1.4 (45–64) reveal that the cause-of-death patterns reported in the main paper hold when we redistribute all the ill-defined deaths to natural causes or external causes, drugs and alcohol mortality for the non-migrants, migrants and the children of migrants. The patterns even hold when we redistribute all ill-defined deaths to one cause among migrants and the children of migrants and the other cause among non-migrants.

Online supplemental file 2 displays all of the primary analysis in the main stratified by sex. At ages 15–44, the all-cause (online supplemental table S2.1) and cause-specific (online supplemental table S2.2) patterns are very consistent across sex. We only report differences among men and women born in Finland (men have higher HRs of all-cause and natural mortality, but not women) and the parent(s) of men and women born in the Baltic States (women have higher all-cause and natural mortality, but not men). At ages 45–64, we also report a good level of consistency. Simultaneously, we see a tendency for men born in Finland, former Yugoslavia, the Baltic States and Other Central & Eastern Europe to report higher HRs of all-cause mortality (online supplemental table S2.3) and natural cause mortality (online supplemental table S2.4)—notably in the minimally adjusted model—when migrant women born in the same countries report lower HRs. Regression-standardised probabilities of death at ages 15–44 (online supplemental table S2.5) and 45–64 (online supplemental table S2.6) are always lower for women.

Online supplemental file 3 reports origin group-specific information about socioeconomic background. Among the children of migrants aged 15–44 in online supplemental table S3.1, although most groups (except Finns and former Yugoslavians) are as highly educated as non-migrants, they are disproportionately concentrated in the lowest disposable income quintile. At older working ages, the situation is more positive—all children of migrant groups are at least as highly educated as non-migrants are. Groups are also more highly represented in the highest disposable income quintile than non-migrants. This is truest of older working age children of migrants with parent(s) born in Central & Southern America, MENAT, Sub-Saharan Africa and other non-Western countries. Among migrants, younger and older working age migrants born in Central & Southern America, MENAT countries, Sub-Saharan Africa and other non-Western countries are disproportionately concentrated in the lowest disposable income quintiles and high shares of migrants aged 15–44 born in MENAT countries and Sub-Saharan Africa have a less than secondary education level. The same is broadly true of other populations, except migrants born in DACH countries, UK, USA, Can, Aus and NZ, and other Western Europe, who are very highly qualified and exhibit similar distributions in disposable income to non-migrants.

## Discussion

Here, we have investigated all-cause, natural and external, drug and alcohol mortality among younger and older working children of migrants and migrants living in Sweden between 1990 and 2023. Among children of migrants, we reported higher all-cause and external, drug and alcohol mortality at younger working ages in all populations (except for DACH countries and other Western Europe). At older working ages, there was little evidence of this higher all-cause mortality—except among children of parent(s) born in Finland, the other Nordic countries and Central & Eastern Europe. Despite this, nearly all older working age children of migrant groups retained a higher risk of mortality from external causes, drugs and alcohol. It was only through the shifting weight of these two cause groups in all-cause mortality over age—from predominantly external causes (at younger working ages) to principally natural causes (at older working ages) that older working age children of migrants did not report high all-cause mortality relative to non-migrants. In contrast, we found universally lower all-cause, natural and external, drug and alcohol mortality among all migrant groups aged 15–44. At ages 45–64, the same remained true of migrants born in other Western Europe, the UK, USA, Can, Aus and NZ, Central & Southern America, MENAT, Sub-Saharan Africa and other non-Western countries, but not of migrants born in other Nordic countries, former Yugoslavia, DACH countries, Baltic States and other Central & Eastern Europe—who had comparable to higher mortality than older working age non-migrants. While adjusting for differences in socioeconomic background emphasised the already lower mortality of most migrant groups, it exerted a salient attenuating effect on the higher mortality of many of the children of migrant populations.

### Previous evidence and what we add

Our results are consistent with previous research studying the mortality of the children of migrants in Sweden and elsewhere. Existing research in Sweden has reported this disparity in mortality risk between migrants and children of migrants at young working ages for many of the groups defined here—highlighting the influential role of suicide and substance misuse mortality among children of migrants^9^ and the powerful attenuating effect of a lower socioeconomic background.^24^ Elsewhere, studies have reported higher mortality among working age male children of migrants (18–64) with Northern African (but not Southern European) origins in France,^12 13^ Turkish and Magrebhan and Sub-Saharan African origins (aged 15–34) in Brussels,^7^ French, Moroccan, Turkish and Sub-Saharan African (but not Italian or Dutch) origins (aged 25–54) in Belgium,^8^ and Black Caribbean, Black Other, and Pakistani and Bangladeshi (but not Indian or Black African) origins (aged 20+) in England and Wales.^11^ Importantly, in all of these studies, the higher mortality risks of the children of migrants contrasted with the lower mortality risks of migrants with the same background. What’s more, these higher mortality risks attenuated after adjusting for disadvantages in socioeconomic background, which were typically measured using education level, income or economic activity, and/or housing. In a sensitivity analysis that tracks coherently with the findings from our analysis, Guillot *et al* split their working age range into 18–44 and 45–64 and found that the all-cause mortality risk of the Northern African group was only elevated at younger working ages.^13^ The paper did not have information on specific causes of death.

What we contribute above and beyond the above studies is a dedicated age perspective, examining mortality risks at younger and older working ages, alongside the provision of cause-specific estimates of mortality from natural causes and external causes, drugs and alcohol. Moreover, while prior studies have focused on relative differences between migrants, the children of migrants and non-migrants, we produce relative (ie, hazard ratios) and absolute (ie, regression-standardised probabilities of death) differences. Importantly, this allows us to contextualise the magnitude of the former in view of the latter. Mortality is a rare event at working ages—while the hazard ratios might be large in certain groups, the regression-standardised probabilities tell us that the absolute impact on mortality is small.

### Interpretation

Why do the all-cause and external, drug and alcohol mortality risks among the children of migrants differ from migrants and non-migrants? We reported a key attenuating role of disadvantaged socioeconomic background on the higher mortality risks of younger working age children of migrants. External mortality has strong links to socioeconomic background because disadvantaged groups, through poverty, inequality, social isolation, a lack of opportunity and neighbourhood segregation, face increased exposure to riskier environments, hazardous work conditions, violent situations and unsafe transport.^1^ So, it is no surprise that adjusting for inequalities in education level and disposable income partially attenuated the higher external, drug and alcohol mortality of younger working age children of migrants. Nevertheless, it is also true that higher risks persisted in many groups after adjustment. Furthermore, persistent, high external, drug and alcohol death risks were also reported among older working age children of migrants—who exhibited favourable distributions in education and disposable income compared with non-migrants. All-cause, natural and external, drug and alcohol mortality was also lower in younger working age migrants before adjustment for socioeconomic background, despite their unfavourable education and disposable income distributions—the well-known Healthy Migrant Paradox.^25^ On the one hand, it may be that, together, education and disposable income do not entirely capture the socioeconomic disadvantages that are most pertinent to external mortality. On the other hand, what other explanations can we seek? Previous research in Sweden has reported an attenuating effect of low childhood socioeconomic background on the young adult mortality of the children of migrants.^10^ These long-arm effects may be operating to generate this persistent, elevated external, drug and alcohol mortality risks among younger and older working-age children of migrants. Moreover, we lack data on what migrants’ lives were like before they moved, but researchers posit that migrants are positively selected on their health and socioeconomic status.^26 27^ With this in mind, migrants may have been socioeconomically advantaged during their time in the country of birth, so their mortality risks may not reflect the higher mortality risks associated with a lower socioeconomic background in Sweden. There is no evidence that the children of migrants benefit in any way from selection effects in their parents—or that children of migrants are selected themselves (as they do not migrate).^1^

We might also consider potential unique psychosocial effects. The children of migrants (unlike migrants) can first encounter racism and discrimination in childhood, notably through education, peer interactions and media and public discourse. They continue to experience it as adults (like migrants) in domains such as workplaces and housing. This earlier exposure matters because racism and discrimination shape stress regulation, identity formation and coping styles when individuals are at their most developmentally vulnerable ages,^28^ and because their influence is recurrent, chronic and cumulative.^29^ A recent review on the impacts of racism and discrimination on developmental outcomes among children of migrants reported lower self-esteem, lower well-being, depressive symptoms and poor overall mental health.^28^ Moreover, acculturative stress among the children of migrants reflects the pressure to adhere to both the cultures of their parent(s) and the culture of the country in which they were born, incorporating family stressors (ie, conflicts related to tradition, increased parental expectations of both academic and workplace achievements), language stressors (eg, among children with parents who do not speak the language of the host country well), and peer stressors (eg, not feeling accepted because of ethnicity).^30^ A higher level of bicultural stress has been reported to be linked to riskier behaviours and a higher rate of depressive symptoms among the children of migrants.^31^,^33^ It may be that these unique psychosocial challenges manifest themselves in a persistent, higher risk of death from external causes, drugs and alcohol.

### Generalisability

Our analysis encompasses the total resident population of Sweden aged 15–64 between 1990 and 2023—our results are fully representative of the situation in Sweden. Although our research is highly consistent with research carried out in other countries, the ability to generalise our findings outside of Sweden might be influenced by factors unique to the country, such as the presence of a universal sociodemocratic welfare state in a country that takes an inclusive multiculturalism approach to ethnic minority status. There may also be differences in its migration history—in the countries that migrants were born in and moved from (eg, sizeable flows from other Nordic countries that are not replicated elsewhere) or the reasons people migrated (eg, with Sweden receiving a large number of refugees relative to its total population size, particularly from MENAT countries and Sub-Saharan Africa). These exemplar factors may interact uniquely with migration background and mortality risk to produce outcomes that are not generalisable to migrant and children of migrant populations elsewhere. Further, we cannot generalise findings on differences in mortality at working ages to infancy and childhood or adult older ages, where the determinants and causes of death are very different to those at working ages.

### Strengths and weaknesses

Strengths of the study include: (1) The use of accurate, reliable and representative total population, administrative register data from Sweden. (2) A focus on younger *and* older working age migrants and children of migrants. (3) The provision of estimates across diverse origins. (4) Implementation of a true competing-risks approach for causes of death. (5) Longitudinal analysis and the use of time-varying covariates to help establish chronology between the predictors and the outcome. (6) The calculation of relative and absolute estimates of death with HRs and regression-standardised probabilities.

Weaknesses of the analysis include that: (1) Our approach is not truly intergenerational. We do not compare migrant parents to their offspring, but concurrent generations of the two groups. As such, the characteristics affecting the mortality risk (ie, observed ones like education and disposable income and unobserved ones like health status, lifestyle behaviours and in-selection effects) of the cohorts of migrants who are the same age as the children of migrants in our analysis may differ from those of their actual migrant parents when they were the same age. Fundamentally, this is a different question to the one we aimed to answer here, but one that *could* be answered with future work and may yield new insights. (2) Although many of the same migrants and children of migrants contribute risk-time to the 15–44 and 45–64 models as they age between 1990 and 2023, the two models also include people who only contribute risk-time to younger or older working ages. Resultantly, while we can definitively conclude that there are differences in all-cause mortality between younger and older working age children of migrants relative to non-migrants, we cannot definitively state that the higher all-cause mortality of younger working age children of migrants will attenuate in older working ages in the future. This is because there may be characteristics unique to younger working age children of migrant cohorts that are not shared by older working age children of migrant cohorts—and vice versa—that could account for differences in mortality risks to non-migrants reported here. (3) Owing to the younger ages and smaller population sizes of the children of migrants, we analysed broad cause-of-death groups that may mask risks from specific causes. For example, while many of the children of migrant populations exhibited a similar to lower mortality risk from natural causes relative to non-migrants, they may face (as yet unknown) elevated risks from specific diseases (eg, from certain cancers) that ‘get lost’ in this broader estimate. (4) Although they are not the focus of the study, we should also consider what potential data and selection effects might overemphasise the extent of the mortality differences between migrants and the children of migrants by artificially lowering the death rates of migrants. These include death under-coverage,^34^ population over-coverage,^35^ age misreporting^36^ and a salmon bias effect. Deaths abroad are captured in Swedish registers (even if a cause-of-death is not always reported) and there should be very little to no death under-coverage.^34^ Work in Sweden additionally demonstrates that while over-coverage is higher in migrants than non-migrants, it only introduces a minimal amount of bias into mortality comparisons between the two groups,^3^ which is consistent with work elsewhere.^37^ There have been no explicit studies of age misreporting in Sweden, but studies from elsewhere only start to see distortions at advanced ages (typically 60+) due to an accumulation of errors over time, small population counts and higher mortality risks at oldest ages.^36 38^ Finally, there is counteracting evidence of a salmon bias effect in Sweden, where migrants with lower, moderate and high disease comorbidity had lower emigration risks compared with migrants without comorbidities.^39^ The same pattern was reported in Denmark too, likely owing to the availability of a universal, tax-financed, free access healthcare system.^39 40^ Such factors cannot explain the mortality risks of the children of migrants. Based on the evidence, they are also unlikely to completely explain the low mortality risks of migrants.

## Conclusions and recommendations

Our main finding was one of persistent and elevated external, drug and alcohol mortality among younger and older working age children of migrants in many origin groups that also acted as the driving force behind the higher all-cause mortality of younger working age children of migrants. This contrasted with typically low all-cause and external, drug and alcohol mortality among younger and older working age migrants with the same origins. We therefore call for interventions that seek to address the higher risks of external, drug and alcohol mortality among children of migrants. Given the powerful attenuating effect of socioeconomic background on all-cause mortality, we call for interventions in areas of life including education, the labour market, housing and welfare that equalise access, support and opportunities to higher education, better paid and more stable jobs, a higher standard of housing and welfare between adult children of migrants and non-migrants. Equally, most children of migrant groups continued to exhibit higher external, drug and alcohol mortality even after having adjusted for disadvantages in education and income. This indicates that targeted interventions into external, drug and alcohol deaths among children of migrants might be necessary. Although current national policies to reduce suicide^41^ and drug misuse deaths^42^ exist that adopt a population-wide approach and cite specific socioeconomic risk factors, there is no mention of the children of migrants, and these programmes may not be reaching this vulnerable population. Going forward, interventions could include culturally-bespoke and community/family-centred programmes that address mental health (eg, collaborative care, psychoeducation, case management, and integrated care and colocation of services),^43^ drug and alcohol use (eg, harm reduction, treatment engagement and access interventions),^44^ violence intervention and mediation (eg, the ‘Cure Violence’ programme^45^) and peer mentoring (eg, one-to-one and/or group-focused initiatives led by people with similar origins).^46^ External mortality *is* preventable (ie, effective interventions can reduce risk before a death occurs) and avoidable (ie, deaths need not occur if systems effectively intervene). Sweden should make it a public health priority to ameliorate the external, drug and alcohol mortality risks of the children of migrants born and living in the country.

## Supplementary material

10.1136/bmjph-2025-003540 online supplemental file 1

10.1136/bmjph-2025-003540 online supplemental file 2

10.1136/bmjph-2025-003540 online supplemental file 3

## Data Availability

Data may be obtained from a third party and are not publicly available.
