# Supplementary material for: Younger adults are not alright, but older adults are? Examining mortality disparities among the children of migrants aged 15–44 and 45–64 in Sweden, 1990–2023: a total-population cohort study
Source: BMJ Public Health. 2026 Mar 26;4(1):e003540. doi: 10.1136/bmjph-2025-003540 (PMC13034282; doi:10.1136/bmjph-2025-003540)
Supplement: online supplemental file 1 [file bmjph-4-1-s001.docx]

**Table S1.1.** Distribution of ill-defined causes of death across main variables.

*Source: author’s calculations based upon Swedish register data collection REFU-GEN.*

**Table S1.1 (cont.)**

*Source: author’s calculations based upon Swedish register data collection REFU-GEN.*

**Table S1.2.** Hazard ratios and regression-standardised cumulative probabilities of death from ill-defined causes derived from fully-adjusted models 1d and 2d.

 *Source: author’s calculations based upon Swedish register data collection REFU-GEN.*

**Table S1.3.** Impact of the redistribution of ill-defined causes-of-death on hazard ratios of external, drug, and alcohol and natural mortality among 15-44 year olds.

*Source: author’s calculations based upon Swedish register data collection REFU-GEN.*

**Table S1.4.** Impact of the redistribution of ill-defined causes-of-death on hazard ratios of external, drug, and alcohol and natural mortality among 45-64 year olds.

*Source: author’s calculations based upon Swedish register data collection REFU-GEN.*
