# Supplementary material for: Younger adults are not alright, but older adults are? Examining mortality disparities among the children of migrants aged 15–44 and 45–64 in Sweden, 1990–2023: a total-population cohort study
Source: BMJ Public Health. 2026 Mar 26;4(1):e003540. doi: 10.1136/bmjph-2025-003540 (PMC13034282; doi:10.1136/bmjph-2025-003540)
Supplement: online supplemental file 2 [file bmjph-4-1-s002.docx]

**Table S2.1.** Hazard ratios of sex-adjusted and sex-specific all-cause mortality among migrants, children of migrants, and non-migrants aged 15-44, 1990-2023.

*Source: author’s calculations based upon Swedish register data collection REFU-GEN.*

Notes: **a** models adjust for sex (in case of first two columns), birth year and the target population; **b** models further adjusted for civil status, education, and disposable income.

**Table S2.2.** Hazard ratios of sex-adjusted & sex-specific cause-specific mortality among migrants, children of migrants, and non-migrants aged 15-44, 1990-2023.

*Source: author’s calculations based upon Swedish register data collection REFU-GEN.* Notes: **a** models adjust for sex (in case of first two columns), birth year and the target population; **b** models further adjusted for civil status, education, and disposable income.

**Table S2.3.** Hazard ratios of sex-adjusted and sex-specific all-cause mortality among migrants, children of migrants, and non-migrants aged 45-64, 1990-2023. *Source: author’s calculations based upon Swedish register data collection REFU-GEN.*

Notes: **a** models adjust for sex (in case of first two columns), birth year and the target population; **b** models further adjusted for civil status, education, and disposable income.

**Table S2.4.** Hazard ratios of sex-adjusted, sex-specific cause-specific mortality among migrants, children of migrants, and non-migrants aged 45-64, 1990-2023. *Source: author’s calculations based upon Swedish register data collection REFU-GEN.*

Notes: **a** models adjust for sex (in case of first two columns), birth year and the target population; **b** models further adjusted for civil status, education, and disposable income.

**Table S2.5.** Regression-standardised cumulative probabilities of death by age 44 and age 64 among male migrants, children of migrants, non-migrants, 1990-2023.  *Source: author’s calculations based upon Swedish register data collection REFU-GEN.*

Notes: cumulative probabilities are regression-standardised from **b** models that adjust for age (as survival baseline), birth year, civil status, education, and disposable income.

**Table S2.6.** Regression-standardised cumulative probabilities of death by age 44 and 64 among female migrants, children of migrants, non-migrants, 1990-2023.  *Source: author’s calculations based upon Swedish register data collection REFU-GEN.*

Notes: cumulative probabilities are regression-standardised from **b** models that adjust for age (as survival baseline), birth year, civil status, education, and disposable income.
