# Supplementary material for: Younger adults are not alright, but older adults are? Examining mortality disparities among the children of migrants aged 15–44 and 45–64 in Sweden, 1990–2023: a total-population cohort study
Source: BMJ Public Health. 2026 Mar 26;4(1):e003540. doi: 10.1136/bmjph-2025-003540 (PMC13034282; doi:10.1136/bmjph-2025-003540)
Supplement: online supplemental file 3 [file bmjph-4-1-s003.docx]

**Table S3.1.** Distributions of education level and disposable income over age and origins among migrants, the children of migrants, and non-migrants, 1990-2023.

*Source: author’s calculations based upon Swedish register data collection REFU-GEN.*

Notes: percentages derived from descriptive *stptime* person-years at risk calculations.
